# Initiation and interruption in intravenous bisphosphonate therapy among patients with multiple myeloma in the United States

**DOI:** 10.1002/cam4.1869

**Published:** 2018-12-08

**Authors:** Leah J. McGrath, Rohini K. Hernandez, Robert Overman, Diane Reams, Alexander Liede, M. Alan Brookhart, Elizabeth O’Donnell

**Affiliations:** ^1^ NoviSci Durham North Carolina; ^2^ Amgen Inc. Thousand Oaks California; ^3^ Massachusetts General Hospital, Harvard Medical School Boston Massachusetts

**Keywords:** bisphosphonates, electronic health records, medical record linkage, multiple myeloma, zoledronic acid

## Abstract

**Background:**

Prior to 2018, intravenous bisphosphonates (IV BPs) were the only therapies recommended to prevent skeletal‐related events for patients diagnosed with multiple myeloma (MM). We examined patterns of IV BP initiation and interruption among patients with newly diagnosed MM (NDMM) in the United States.

**Methods:**

Electronic health records linked to administrative health insurance claims were used to identify adults with NDMM between 1 January 2011 and 30 April 2016. Patients were excluded for recent IV BP use or concurrent cancer. The incidences of IV BP initiation and interruption were estimated using competing risk regression. A generalized linear model was used to estimate risk factors for treatment initiation and interruption.

**Results:**

Among the 547 patients with NDMM, 64% initiated MM therapy within 30 days of diagnosis. By one year, 65% (95% CI: 59, 70) of patients with appropriately timed anti‐MM therapy had initiated an IV BP. Zoledronic acid was the most commonly initiated IV BP. Patients with Stage III MM were more likely to initiate an IV BP (adjusted risk difference (RD): 6.3; 95% CI: 2.7, 10.1), while those with eGFR <30 mL/min were less likely to initiate (RD: −9.7; 95% CI: −13.8, −5.8). Of the 264 patients who initiated an IV BP, 77% (95% CI: 71, 82) experienced an interruption within one year. Patients on concurrent anti‐MM therapy were less likely to experience an interruption in IV BP therapy.

**Conclusions:**

Many patients with NDMM do not initiate IV BPs, particularly those with renal complications. Interruptions of IV BPs were common.

## 1 INTRODUCTION

Osteolytic lesions occur in up to 90% of patients diagnosed with multiple myeloma (MM)^1^, ^2^ and increase the risk for skeletal‐related events (SREs), such as pathologic fractures, spinal cord compression, requirement for surgery, or palliative radiotherapy to bone.^3^ Intravenous bisphosphonates (IV BPs) can be administered to manage bone disease and prevent SREs.^4^ Historically, zoledronic acid and pamidronate were the only approved therapies in the United States (US) for preventing SREs due to MM. Both IV pamidronate and zoledronic acid have been shown to reduce SREs vs placebo or older IV BPs.^5^, ^6^ The International Myeloma Working Group recommends initiation of IV BPs in patients diagnosed with MM, with or without detectable osteolytic bone lesions on conventional radiography, who are receiving antimyeloma therapy, as well as patients with osteoporosis or osteopenia resulting from MM.^7^ It is recommended that IV BPs be administered at 3 to 4‐week intervals for all patients with active MM. In 2018, denosumab,^8^ a human monoclonal antibody against receptor activator of nuclear factor kappa‐B ligand that inhibits osteoclast formation and decreases bone resorption and induced bone destruction was approved in the US and EU for SRE prevention.

Relatively little is known about patterns of use of IV BPs in patients with newly diagnosed MM (NDMM) in routine clinical practice. Additionally, there is no clinical consensus on whether to continue, reduce, or stop IV BPs among patients who achieve a good response. As the treatment options for preventing SREs in this population are evolving, it is important to understand how IV BPs are being used in contemporary practice and identify specific areas of unmet need. The goal of this study is to describe IV BP treatment patterns in patients with NDMM who were receiving care in oncology/hematology clinics in the US.

## 2 METHODS

### 2.1 Data source

We used electronic health record (EHR) data from oncology/hematology clinics across the US (Flatiron Health, Inc., New York, NY, USA) linked to MarketScan® employer‐based and Medicare Supplemental administrative health insurance claims databases (Truven Health Analytics, Inc., Ann Arbor, MI, USA).

The Flatiron EHR includes data from more than 255 cancer clinics and 2330 clinicians across the US, which includes more than 1.3 million active cancer patients. Diagnoses, laboratory results, and medications administered within the cancer clinic are collected. The MarketScan database captures healthcare claims data for privately insured individuals (<65 years) and individuals with Medicare Supplemental insurance (≥65 years). Available data include inpatient and outpatient diagnoses, procedures, and medications, which are identified via International Classification of Diseases, clinical modification, ninth or tenth revision (ICD‐9‐CM and ICD‐10‐CM), Healthcare Common Procedure Coding System (HCPCS), National Drug Codes (NDC), and/or Current Procedural Terminology (CPT) codes. The EHR was linked to the MarketScan data at the patient level for patient records found in both data sources, although timing of enrollment in each data source was not considered during the linkage process.

### 2.2 Cohort selection

Patients were eligible for inclusion if they were aged 18 years or older and had a new MM diagnosis (ICD9, 203.00; ICD10, C90.00) between 1 January 2011 and 30 April 2016. Patients were excluded if they received an IV BP in the six months prior to the MM diagnosis, had concurrent cancer at a second primary site in the 12 months prior to diagnosis, or did not have a healthcare encounter in the 30 days following the diagnosis to ensure they were “active” in the EHR. Patients were additionally required to have continuous enrollment in MarketScan in the six months before the MM diagnosis.

### 2.3 Outcomes and follow‐up

Two cohorts were created to examine IV BP treatment initiation. All patients with NDMM entered these cohorts on the date of their diagnosis. Patients were then separated into a subgroup who received antimyeloma therapy within 30 days before or after their diagnosis (appropriately timed therapy) vs patients who did not receive antimyeloma therapy during this period (untreated). IV BP initiation was defined as the first administration of either pamidronate or zoledronic acid following a new diagnosis of MM. IV BP administrations were identified using either data source. If there were discrepancies in the dates of administration between the two data sources, the earliest date was used. To examine IV BP interruption, the cohort included all patients from the larger cohort of patients with NDMM who initiated an IV BP. Interruption was defined as having an absence of the specific IV BP treatment in a 45‐day interval since the last IV BP treatment. A 45‐day interval was chosen to capture the recommended administration time window for IV BPs (once per 28 days) plus a short grace period. As a sensitivity analysis, we extended the treatment interruption window to a 90‐day interval to allow for an extended‐dosing schedule.

The start of follow‐up (ie, the index date) was the date of the new MM diagnosis (treatment initiation analysis) or the date of IV BP treatment initiation (treatment interruption analysis). Each patient was followed from the index date until the occurrence of the event of interest, death, 30 June 2016, stem cell transplant, or lost to follow‐up defined as 90 days without a healthcare encounter in the EHR or MarketScan disenrollment, whichever came first.

### 2.4 Covariates

Covariates were identified using both data sources, and baseline characteristics were updated at the index date for the nested cohort. Baseline chronic comorbidities were defined using all available data. Baseline laboratory tests were measured in the 60 days prior to the MM diagnosis. If there were multiple test results during that period, the most recent result was used. MM stage at diagnosis was determined using the EHR at any point before or within 30 days after the NDMM diagnosis. If stage was missing, it was derived using serum beta‐2 microglobulin (B2M) and serum albumin.^9^ Time‐varying covariates (lab results, comorbidities, other therapies, and SREs) were identified: (a) during the 30‐day period following the diagnosis of MM and during each 30‐day interval thereafter (for risk factors of treatment initiation); and (b) during the 45‐day period following treatment initiation and during each 45‐day interval thereafter (for risk factors of treatment interruption).

### 2.5 Statistical analysis

Descriptive statistics were calculated for baseline covariates at the time of the new MM diagnosis (for the IV BP treatment initiation analysis) and recalculated at the initiation of an IV BP (for the treatment interruption analysis). The cumulative incidences of treatment initiation and interruption were estimated using Fine‐Gray regression models that accounted for the competing risk of death.^10^ To assess risk factors associated with treatment initiation and interruption, we estimated the absolute difference in monthly risk of each outcome using multivariable repeated measures generalized models with an identity link function that provided estimates of risk differences. Asymptotically correct 95% confidence intervals (CIs) were obtained using a parametric bootstrap.^11^ Risk factors for each outcome were drawn from the interval prior to the interval in which the outcome occurred, as well as all prior intervals and the baseline period. This study was approved by the Chesapeake Institutional Review Board. All statistical analyses were performed using R software, version 3.4.0 (R Foundation for Statistical Computing, Vienna, Austria).

## 3 RESULTS

**Figure 1:**
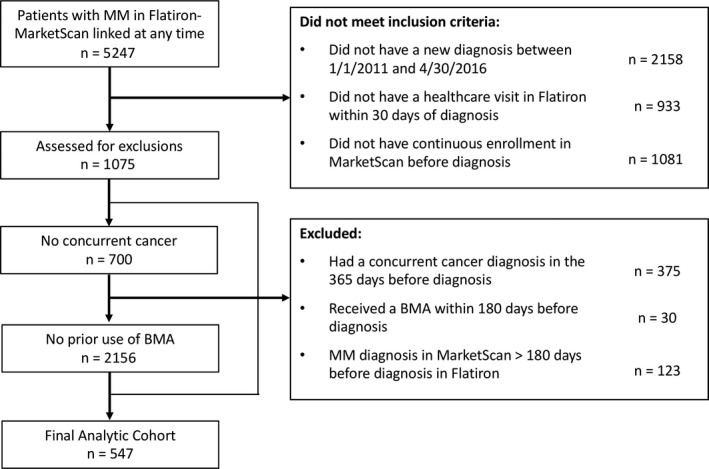
Study flow diagram

Between January 2011 and April 2016, 5247 patients with MM were identified using the Flatiron EHR. Of those, 547 patients met eligibility criteria for inclusion (Figure 1). Of patients with a new diagnosis of MM, 348 (64%) initiated appropriately timed treatment for MM within 30 days of their diagnosis. Among those who initiated appropriately timed MM treatment, 51% were 65 years or older, 58% were male, 66% were White, and 24% had a history of an SRE (Table 1). There was a higher prevalence of Stage I MM (41% vs 16%) and a higher median beta‐2 microglobulin (4.2 vs 2.6) among patients who were untreated with anti‐MM therapy compared to those who received appropriately timed MM therapy. Furthermore, patients who were initially untreated with anti‐MM therapy had a lower prevalence of renal disease (39% vs 51%) and SREs (16% vs 24%) compared with patients who did receive treatment. Seventy‐five (14%) patients with NDMM received a stem cell transplant during follow‐up.

**Table 1:** Baseline characteristics of patients with multiple myeloma at multiple myeloma diagnosis and intravenous bisphosphonate initiation

Variable	At NDMM Diagnosis				At IV BP initiation	
	Appropriately timed anti‐MM therapy^a^		Untreated with anti‐MM therapy			
	N or Median	% or IQR	N or Median	% or IQR	N or Median	% or IQR
Total	348	100	199	100	264	100
Demographics						
Age, years						
18‐39	7	2.0	3	1.5	3	1.1
40‐49	27	7.8	14	7.0	17	6.4
50‐64	137	39.4	76	38.2	112	42.4
65+	177	50.9	106	53.3	132	50.0
Male sex	203	58.3	105	52.8	151	57.2
Race						
White	230	66.1	120	60.3	176	66.7
Black	62	17.8	38	19.1	44	16.7
Asian	2	0.6	1	0.5	2	0.8
Other	18	5.2	10	5.0	11	4.2
Missing	36	10.3	30	15.1	31	11.7
Practice type						
Academic	22	6.3	23	11.6	14	5.3
Community	326	93.7	176	88.4	250	94.7
Stage at multiple myeloma diagnosis^b^						
Stage I	56	16.1	82	41.2	60	22.7
Stage II	105	30.2	35	17.6	76	28.8
Stage III	82	23.6	5	2.5	69	26.1
Missing	105	30.2	77	38.7	59	22.3
Insurance payer						
Commercial health plan	102	29.3	56	28.1	82	31.1
Medicare	17	4.9	10	5.0	11	4.2
Multiple	134	38.5	76	38.2	101	38.3
Other	27	7.8	10	5.0	23	8.7
Missing	68	19.5	47	23.6	47	17.8
Comorbidities^c^						
Diabetes	133	38.2	72	36.2	95	36.0
Chronic obstructive pulmonary disease and asthma	140	40.2	69	34.7	103	39.0
Osteoporosis	78	22.4	54	27.1	67	25.4
Coronary artery disease	165	47.4	91	45.7	116	43.9
Liver disease	44	12.6	25	12.6	39	14.8
Renal disease or impairment	179	51.4	77	38.7	107	40.5
Neuropathy	170	48.9	81	40.7	129	48.9
Frailty indicators^c^						
Congestive heart failure	139	39.9	64	32.2	98	37.1
Decubitus ulcer	20	5.7	13	6.5	20	7.6
Difficulty walking	46	13.2	38	19.1	46	17.4
Walker	29	8.3	20	10.1	40	15.2
Rehabilitation services	310	89.1	178	89.4	238	90.2
Vertigo	95	27.3	48	24.1	73	27.7
Other frailty indicators^d^	27	7.8	21	10.6	32	12.1
History of SREs^c^						
Any SRE	83	23.9	31	15.6	97	36.7
Pathological fracture	59	17.0	19	9.5	71	26.9
Spinal cord compression	10	2.9	1	0.5	9	3.4
External beam radiation therapy	8	2.3	3	1.5	20	7.6
Bone surgery	36	10.3	17	8.5	44	16.7
Other treatments						
Immunomodulating agent	187	53.7	0	0.0	134	50.8
Proteasome inhibitors	278	79.9	0	0.0	196	74.2
Monoclonal antibodies	2	0.6	0	0.0	1	0.4
Chemotherapy	81	23.3	0	0.0	61	23.1
Colony stimulating factor	7	2.0	0	0.0	8	3.0
Opioids	161	46.3	47	23.6	147	55.7
Glucocorticoids	279	80.2	23	11.6	200	75.8
Labs						
Serum albumin, g/dL						
Normal: 3.5‐5 g/dL	140	40.2	91	45.7	144	54.5
Low <3.5 g/dL	65	18.7	21	10.6	84	31.8
High >5 g/dL	4	1.1	4	2.0	3	1.1
Serum calcium, mg/dL						
Normal: 9.1‐10.7 mg/dL	122	35.1	90	45.2	113	42.8
Hypocalcemia: <9.1 mg/dL	73	21.0	26	13.1	102	38.6
Hypercalcemia: ≥10.8 mg/dL	14	4.0	2	1.0	19	7.2
Beta‐2 microglobulin						
Median (IQR)	10.8	(9.7, 12.3)	12.4	(10.8, 13.4)	10.8	(9.8, 12.0)
Missing	118	33.9	71	35.7	28	10.6
eGFR, mL/min						
<30	33	9.5	8	4.0	8	3.0
30 to <60	55	15.8	27	13.6	50	18.9
≥60	94	27.0	64	32.2	146	55.3

eGFR, estimated glomerular filtration rate; IV BP, intravenous bisphosphonates; MM, multiple myeloma; NDMM, newly diagnosed MM; SRE, skeletal‐related event

a Early MM treatment is defined as any MM‐specific therapy within the 30 d before or after NDMM diagnosis.

b MM stage was determined directly from the EHR or using values for beta‐2 microglobulin and serum albumin as defined in the International Staging System

c Chronic comorbidities and SREs were assessed using all available data prior to the index date.

d Other frailty indicators included oxygen use, paralysis, weakness, or wheelchair use.

Following a NDMM diagnosis, 264 patients initiated an IV BP and were followed for treatment interruption (Table 1). Demographics and the use of MM therapy for IV BP‐treated patients were similar to the starting cohort measured at NDMM diagnosis, but the prevalence of SREs was higher (37%), indicating that patients were experiencing SREs during follow‐up (Table 1).

### 3.1 IV BP initiation

**Figure 2:**
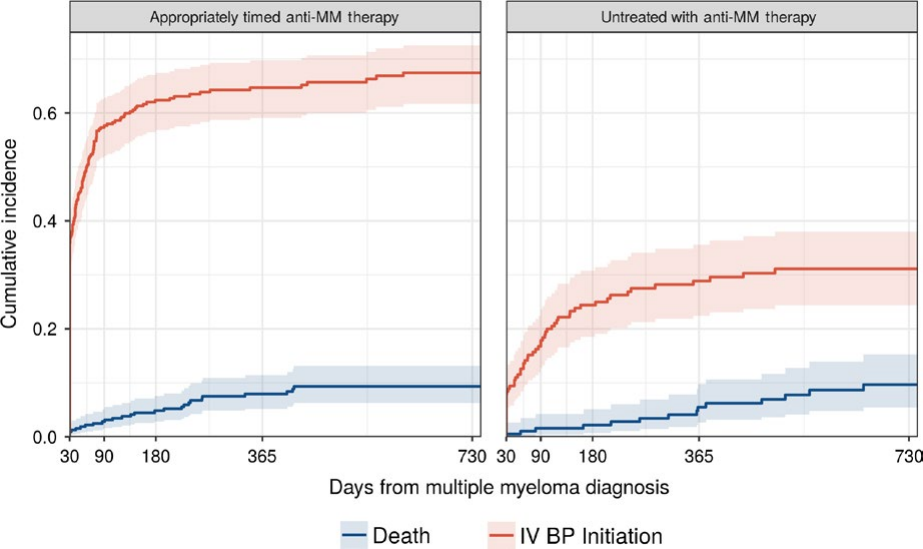
Cumulative incidence of intravenous bisphosphonate initiation by presence or absence of appropriately timed multiple myeloma therapy (within 30 d of multiple myeloma diagnosis) among patients with multiple myeloma in the United States

The cumulative incidence of IV BP treatment initiation after diagnosis of NDMM among patients with appropriately timed anti‐MM treatment was 36% (95% confidence interval [CI]: 31, 41%) at 30 days, 58% (52, 63%) at 90 days, 65% (59, 70%) at one year (Figure 2). Incidence of initiation among patients who were not treated with appropriately timed anti‐MM treatment was much lower (29% at 1 year). Among those who initiated an IV BP, 95% received zoledronic acid, while only 5% received pamidronate, and 9% received only one dose before discontinuing.

**Figure 3:**
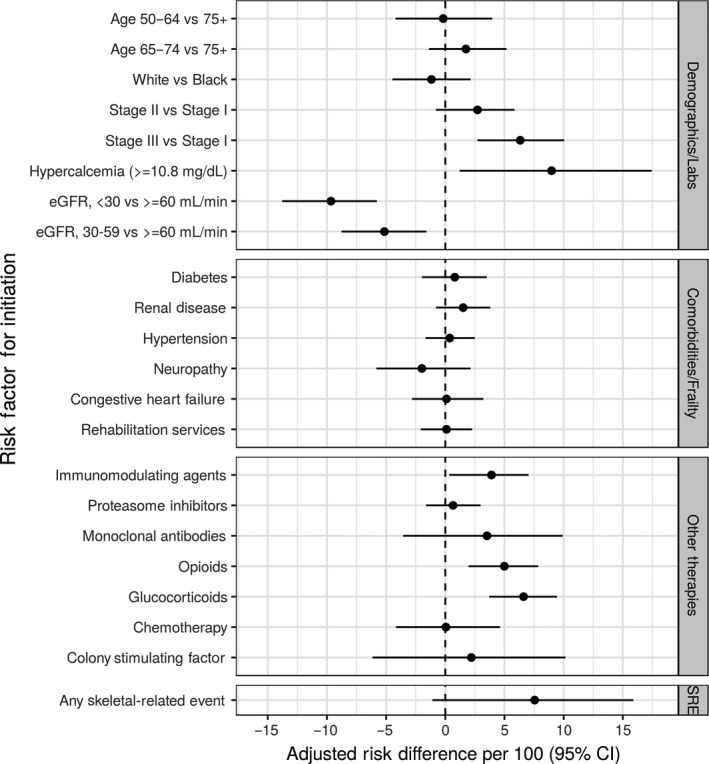
Adjusted risk difference (aRD) estimates per 100 for variables associated with intravenous bisphosphonate initiation

Patients with Stage III MM had 6.3 additional IV BP initiation events per 100 people (95% CI: 2.7, 10.1) compared with Stage I patients. Receipt of opioids (adjusted risk difference [RD]: 5.0; 95% CI: 2.0, 7.9) was also positively associated with IV BP initiation. While having a previous SRE was associated with IV BP initiation, the confidence interval was wide (RD: 7.6; 95% CI: −5.8, 15.9). Additionally, severe renal impairment, defined as eGFR <30 mL/min, or moderate renal impairment, defined as eGFR 30‐60 mL/min, were negatively associated with IV BP treatment (RD: −9.7 and −5.2, respectively; Figure 3).

### 3.2 IV BP interruption

**Figure 4:**
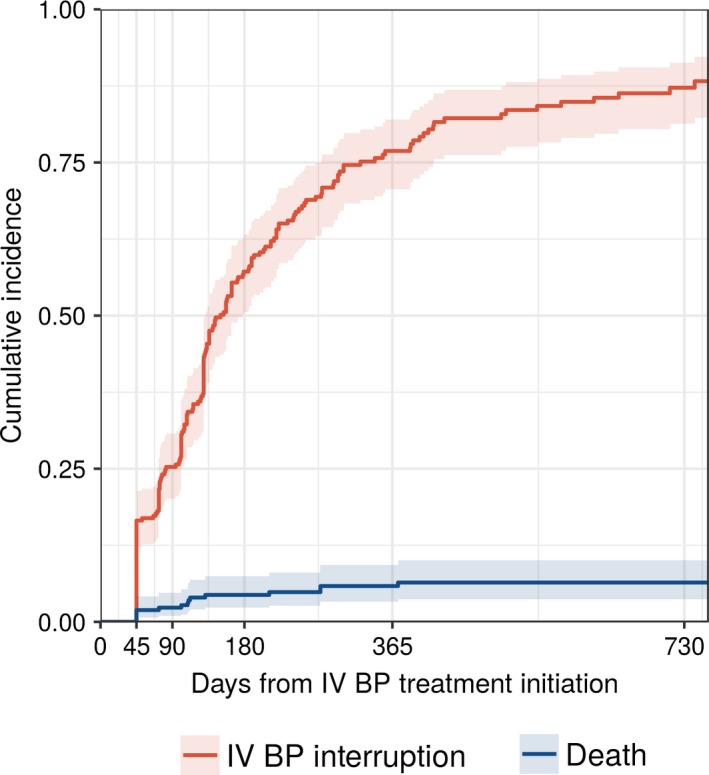
Cumulative incidence of intravenous bisphosphonate interruption among patients with multiple myeloma in the United States

The cumulative incidence of treatment interruption after initiation was 17% (95% CI: 12, 21%) at 45 days, 25% (95% CI: 20, 31%) at 90 days, 77% (95% CI: 71, 82%) at one year (Figure 4). When using a 90‐day treatment interruption definition, the cumulative incidence at one year was 48% (95% CI: 42, 55%).

**Figure 5:**
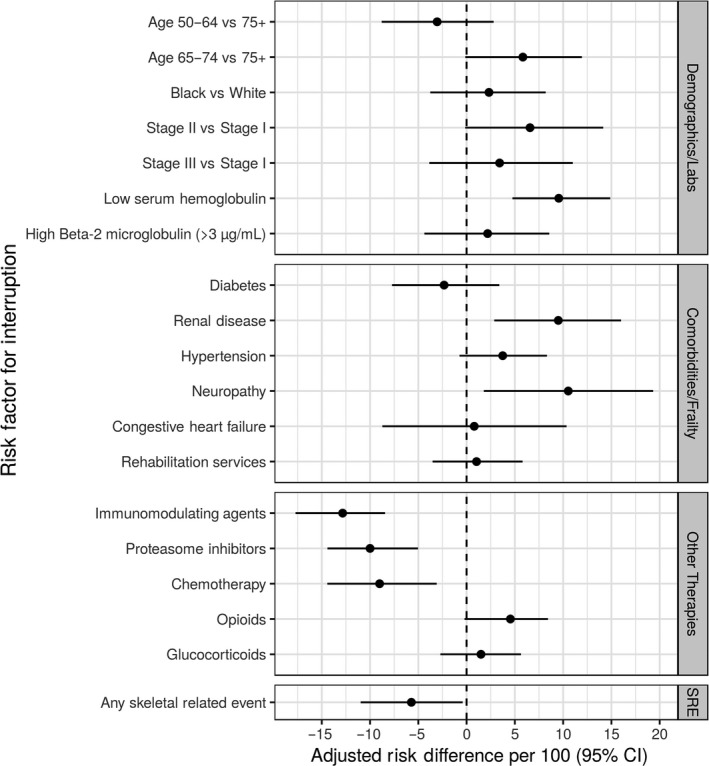
Adjusted risk difference (aRD) estimates per 100 for variables associated with intravenous bisphosphonate interruption

Intravenous bisphosphonates interruption was more likely among patients with renal disease, low serum hemoglobin, and patients prescribed opioids. IV BP interruption was less likely among patients receiving immunomodulating agents, proteasome inhibitors, and chemotherapy (Figure 5).

## 4 DISCUSSION

Prior to 2018, IV BPs were the only recommended therapy for patients diagnosed with MM, with or without detectable osteolytic bone lesions receiving antimyeloma therapy, as well as patients with osteoporosis or osteopenia resulting from myeloma. In this study, cohort of patients diagnosed in routine clinical care, 35% of patients actively managing their MM did not receive an IV BP within one year of MM diagnosis. More than 70% of patients who did not receive anti‐MM therapy within 30 days after MM diagnosis also did not receive an IV BP. This population could include patients with MGUS and asymptomatic MM, who are not recommended to receive IV BPs unless osteoporosis is present, or could represent patients who experience treatment delay in response to nonemergent symptoms.^7^ Among patients who started an IV BP, more than 75% interrupted treatment (ie, experienced a >45‐day gap) within a year of starting. However, only about half interrupted treatment when interruption was defined using a 90‐day treatment gap, suggesting that physicians may be extending the dosing period.

The estimate of IV BP initiation in our study (65%) was similar to a recent study using EHR data, in which 63% of patients with MM received at least one IV BP administration.^12^ Another study using data from older years showed that only 39% received an IV BP.^13^ This estimate is similar to our study if patients were not stratified by appropriately timed anti‐MM treatment status. We saw a much lower use of IV BPs among patients who did not receive anti‐MM therapy within 30 days after diagnosis.

Having a history of renal disease was common in this cohort (51% at NDMM diagnosis), which could affect administration of IV BPs. Zoledronic acid, the most commonly used IV BP, has been shown to cause renal toxicity and is not recommended among patients with severe renal impairment.^14^ Potential renal impairment may dissuade physicians from using IV BPs among patients with renal complications. This pattern was seen in our data, as both severe and moderate renal impairment were negatively associated with IV BP treatment initiation, and patients with renal disease were more likely to experience interruptions in treatment. The approval of denosumab, which has been shown to be noninferior to zoledronic acid,^15^ may provide clinicians with additional treatment options among patients with renal complications as it is neither renally cleared nor nephrotoxic.

One of the strongest risk factors for IV BP initiation was a history of a SRE. Although the confidence interval was wide, this finding suggests that IV BPs may be used in some cases to prevent a subsequent SRE, rather than to prevent the first SRE. Additionally, patients with more advanced MM or those prescribed opioids were more likely to receive an IV BP. It is likely that these patients had more advanced bone disease and/or were experiencing bone pain. There are several reasons why treating bone disease may benefit patients with MM. Treating bone disease can not only improve quality of life, but also increases overall survival.^16^ New therapies for treating bone disease are also showing promise in treating the cancer itself.^17^, ^18^

Patients who received appropriately timed anti‐MM therapy within 30 days of MM diagnosis were less likely to experience an interruption in IV BP treatment than patients who did not receive anti‐MM therapy during this period. The dosing schedules for many anti‐MM therapies coincide with the dosing schedule for IV BPs, which may explain better patient adherence.

There are several limitations to consider when evaluating the results of this study. The data from the EHRs arose primarily from community‐based oncology clinics, and therefore may not be generalizable to other cancer treatment settings. We were unable to capture information on bone lesions (eg, number, location, severity) or dental disease or procedures, both of which could be associated with treatment initiation. Although lab results and functional status (measured via ECOG assessment) were available in the EHR, they were missing for a large proportion of the population at baseline. This limited the ability to discern whether there was an association between these variables and initiation or interruption. However, we maximized the data that was available by updating these variables throughout follow‐up in a time‐dependent manner.

Data from routine clinical practice suggests that patients with NDMM initiate IV BP therapy less frequently than expected, even though these therapies have been shown to be effective in preventing SREs. The majority of patients who do initiate therapy, experience a treatment interruption within the first year after starting treatment. Patterns of treatment may change with the introduction of denosumab in the US beginning in January 2018 and should be re‐evaluated after sufficient follow‐up time elapses.

## CONFLICT OF INTEREST

MAB has received investigator‐initiated research funding from the NIH and through contracts with the AHRQ's DEcIDE program and the PCORI. Within the past three years, he has received research support from Amgen and AstraZeneca and has served as a scientific advisor for Amgen, Merck, GSK, UCB, Genentech, TargetPharma, and RxAnte. MAB owns equity in NoviSci, a data sciences company. RH and AL are employees of Amgen Inc and have stock ownership in Amgen Inc. EO participated on an advisory board for Amgen. LM, RO, DR have no conflict of interests.
